# Depression among AIDS-orphaned children higher than among other orphaned children in southern India

**DOI:** 10.1186/1752-4458-8-13

**Published:** 2014-04-08

**Authors:** SG Prem Kumar, Rakhi Dandona, G Anil Kumar, SP Ramgopal, Lalit Dandona

**Affiliations:** 1 Public Health Foundation of India, ISID Campus, 4, Institutional Area, Vasant Kunj, New Delhi 110 070, India; 2 Institute for Health Metrics and Evaluation, University of Washington, Seattle, WA, USA

**Keywords:** HIV, AIDS, Orphaned children, Mental health, Depression, India

## Abstract

**Background:**

Systematic data on mental health issues among orphaned children are not readily available in India. This study explored depression and its associated risk factors among orphaned children in Hyderabad city in south India.

**Methods:**

400 orphaned children drawn equally from AIDS and non-AIDS orphan groups aged 12–16 years residing in orphanages in and around Hyderabad city in southern India were recruited to assess depression and associated risk factors using the Center for Epidemiologic Studies-Depression Scale (CES-DC). Variation in the intensity of depression was assessed using multiple classification analysis (MCA).

**Results:**

397 (99%) orphans provided complete interviews in the study of whom 306 (76.5%) were aged 12 to 14 years, and 206 (51.8%) were paternal orphans. Children orphaned by AIDS were significantly more likely to report being bullied by friends or relatives (50.3%) and report experiencing discrimination (12.6%) than those orphaned due to other reasons (p < 0.001). The overall prevalence of depression score >15 with CES-DC was 74.1% (95% CI 69.7-78.4) with this being significantly higher for children orphaned by AIDS (84.4%, 95% CI 79.4 – 89.5) than those due to other reasons (63.6%, 95% CI 56.9 – 70.4). Mean depression score was significantly higher for children orphaned by AIDS (34.6) than the other group (20.6; p < 0.001). Among the children orphaned by AIDS, the bulk of depression score was clustered in 12–14 years age groups whereas in the children orphaned by other reasons it was clustered in the 15–16 years age group (p = 0.001). MCA analysis showed being a child orphaned by AIDS had the highest effect on the intensity of depression (Beta = 0.473).

**Conclusions:**

Children orphaned by AIDS had significantly higher depressive symptoms than the other orphaned children. These findings could be used for further planning of mental health interventions to meet the mental health needs of orphaned children, that could include preventive, diagnostic and treatment services.

## Introduction

Globally, policy makers are struggling to find care solutions for an estimated 153 million children who have had at least one parent die [1]. High mortality among young adults from conditions such as HIV/AIDS, malaria, tuberculosis, pregnancy complications, and natural disasters are responsible for the large and increasing number of orphans globally with the south and east Asia region estimated to have the largest number of orphans worldwide [2,3]. The orphaned children are impacted by their parental illness followed by their loss, as it limits their access to basic social services and undermines their chances of survival and future [4,5].

It is estimated that more than 20 million were orphaned by AIDS worldwide by 2010 [6]. HIV/AIDS is recognized as a source of mental health issues for young people, orphans and for those caring for orphans [7-9]. Though some recent attempts have been made to address the issues of children affected by AIDS, several issues still remain inadequately addressed especially the mental health of children orphaned by HIV/AIDS (COA) [10-17]. Higher rates of depression and anxiety, stigma and less optimism about future have been reported among COA as compared with non-orphans, and majority of these data are available from Sub-Saharan Africa [10,13,14,18-20].

Reliable estimates of the number of COA is not readily available for India [21,22]. UNICEF estimates that there could be 4 million COA in India but given the high numbers of HIV infected people in India and the lag time between HIV infection and death from AIDS, it is estimated that the number of COA is on the rise [23-26]. India is signatory to the political declaration on HIV/AIDS wherein the government is committed to addressing the issues of COA [27]. Recognizing the need to support the growing orphan population, many non-governmental and faith-based organizations have founded orphanages to care for these children [22]. Data on mental health of these children are not readily available. In this context, we conducted a comparative study of mental health issues among COA and those orphaned due to other disease/condition in urban India in order to contribute to an increased understanding of the mental health of children who have lost their parents, particularly COA.

## Methods

Data for this study were collected from January to March 2012 in 14 orphanages in and around Hyderabad city in southern India. Ethics approval for this study was provided by the Human Ethics committee of the Public Health Foundation of India, New Delhi. Provision was made for referral to a psychologist in the event of distress resulting from interview for children who participated in this study.

### Sample size and selection of participants

Children orphaned due to HIV/AIDS (COA) and those orphaned because of reasons other than HIV/AIDS (COO) aged 12 to 16 years were sampled for this study. An orphan child was defined as a child who had lost one or both parents, and therefore included maternal, paternal and double orphans [28,29]. A scoping exercise was undertaken during November 2011 in and around Hyderabad to identify functional orphanages from where recruitment of children could be done, and to obtain characteristics of orphans housed in these orphanages to assist with sampling (number of orphaned children by age and sex, type of orphan (AIDS or non-AIDS), average duration of stay, predominant language spoken and appropriate time to contact). Based on this scoping exercise, 14 orphanages which had at least 20 orphaned children aged 12 to 16 years were sampled, and these together housed 524 orphaned children. Of these, two orphanages were run by the Government of the Indian state of Andhra Pradesh and the remaining 12 by private non-government organisations (NGOs). AIDS and non-AIDS orphans were housed in exclusive orphanages for each type. A total of 6 orphanages housed COO of which 4 were run by NGOs, and 8 orphanages housed exclusively COA and all these were run by NGOs.

Assuming 80% power to detect a 10% difference in mental health outcomes between COA and COO at the 95% confidence level (95% CI 3.5% - 16.5%), we estimated a total sample size of 167 children from each of AIDS and other orphans children. Assuming a participation rate of 80%, we planned to recruit 400 orphan children to achieve the required number of interviews. We sampled COO in proportion to their estimated number available at each orphanage. However, we sampled all available eligible COA as the numbers of these children were not enough. The children aged 12–16 years who had spent at least 6 months at the orphanage and who could understand at least one of the three languages – Telugu, Hindi or English were considered eligible for the study.

### Data collection

Each potential participant was contacted by an interviewer trained in the study procedures with the assistance of the orphanage staff. The study was explained and informed consent sought for participation. For children aged 12 to 14 years, child assent and the consent from the concerned care-giver/guardian was obtained. All participants had the right to refuse participation or stop interview anytime. Before starting the interview, each participant was narrated a short and simple story to assist the child to understand the context and content of the interview. This story was developed with inputs from a mental health expert with experience in dealing with children. After this narration, the interview was conducted in privacy. Average interview time was 45 minutes following which every participant received a nominal gift as a token of appreciation.

### Measures

The interview documented demographic characteristics of children including age, sex, education, religion, type of orphan, and duration of stay in orphanage. The cause of parental death was documented from the individual details maintained for each child at the orphanage. Mental health related measures documented relevant to this paper included depression, and history of abuse, violence and discrimination. Four rounds of pre-testing of the study instrument were undertaken among orphaned children aged 12–16 years prior to the study by the study investigators in consultation with a psychologist who worked with children. Additionally, inputs from the mental health experts, child counselors and the NGO/orphanage staff were obtained to refine and validate these for the study population. Based on these exercises, certain definitions were simplified, revisions in local language translation for Hindi and Telugu were made, and interview techniques improved. We did not document the HIV status of the study participants.

#### ***Depression***

The Center for Epidemiologic Studies-Depression scale (CES-DC) designed for children aged 6 to 17 years was used to measure depression [19,20]. This scale was translated into the local languages for use by the researchers, and then was back-translated and field-tested to ensure proper readability. As cultural validity was a major concern in translating this scale, the researchers closely collaborated with mental health experts, child counselors and the NGO/orphanage staff to achieve accuracy of cultural understanding and translation. The translation of conceptual meanings of the English version of the CES-DC into local languages was relatively easy and straightforward. The respondents were asked to rate the degree to which they experienced each depression related symptom on a 4-point frequency scale (not at all, a little, some, and a lot), and the possible scores for CES-DC ranged from 0 to 60. A CES‒DC score of 15 or higher has previously been considered suggestive of significant level of depressive symptoms in children and adolescents [19,20].

#### ***Abuse and violence, and discriminationM***

History of abuse and violence by friends or relatives including type of abuse (denial of food; healthcare and other essential needs; denial of financial or property inheritance; and physical, emotional or sexual abuse), experience of abuse in orphanage (denial of basic needs such as food and shelter; verbal abuse; threat of violence; physical beatings; and sexual or mental abuse), and witnessing fights between parents was documented. History of experience with discrimination from friends/relatives and community was also documented.

### Statistical analysis

SPSS version 17.0 was used for data analysis. Descriptive statistics are reported for relevant quantitative variables. Differences between groups (COA and COO) in socio-demographic characteristics, abuse and violence indicators, and discrimination were assessed using Chi-square tests or one-way analysis of variance tests as appropriate.

All analyses used continuous score for depression, and we report prevalence of depression score >15 among these children, a recommended cutoff for clinically significant levels of depressive symptoms [19,20]. Of the 20 items on the CES-DC scale, we also report the items which substantially contributed to the depression burden among the COA and COO groups. Associations of depression with socio-demographic variables and abuse, violence and discrimination indicators were examined using independent simple Fishers-tests. Multivariate analysis of the variation in intensity of depression associated with socio-demographic (age, sex, type of orphan, education, religion and duration of stay in the orphanage) and risk factors for mental health (ever bullied by friends/relatives, ever abused at the orphanage, ever witnessed fights between parents, and ever experienced discrimination) were performed using multiple classification analysis (MCA). We used continuous scale and not the clinical cut-off score of >15 for depression in the multivariate analysis as this score reflects western norms and such norms are not readily available for this study population. 95% confidence interval (CI) is reported as appropriate.

## Results

### Participation and demography

A total of 397 (99.3%) orphaned children aged 12–16 years from 14 orphanages provided interview, of whom 306 (76.5%) were aged 12 to 14 years of age. The median age for both boys and girls was 13 years. The proportion of boys was higher among the COA (63.5%) with a higher portion of girls in the COO (59%). Paternal orphans constituted half of all the children sampled (51.8%) followed by double orphans (30.7%) and maternal orphans (15.1%). Of the total, 397 (99.3%) children were currently in school and 200 (50.6%) belonged to Hindu religion. The average duration of stay in orphanage for COA was 3.2 years (range 0.6 to 8 years) and was 3.6 years (range 0.6 to 12 years) for COO. There was no significant variation in the mean duration of stay in the orphanage for boys (3.3 years) and girls (3.5 years).

### Abuse and violence, and discrimination

COA (N = 100, 50.3%) were significantly more likely to report being bullied by friends or relatives than COO (N = 53, 26.8%; p < 0.001). Similarly, significantly more COA (N = 100, 50.3%) reported witnessing fights between parents when they were alive as compared with COO (N = 71, 35.9%; p = 0.003). A total of 40 (10.1%) orphaned children reported experiencing discrimination ever of whom majority were COA (62.5%; p < 0.001). However, the proportion of orphaned children reported ever experiencing abuse at orphanage was higher among COO (23.2%) than COA (19.1%; p = 0.188).

### Depression

The range of depression score with CES-DC scale was 3 to 56 for COA and 3 to 47 for COO. The prevalence of depression score of >15 was 74.1% (95% CI 69.7 - 78.4), with significantly higher prevalence among COA (84.4%, 95% CI 79.4 – 89.5) than COO (63.6%, 95% CI 56.9 – 70.4) (p < 0.001). Table 1 shows the mean and median depression score with select variables for the orphaned children. Overall, mean depression score was higher for COA (34.6) than COO (20.6). In both the groups, the mean depression score was significantly higher among those who reported being bullied or ill-treated by friends or relatives and those who reported experiencing discrimination from friends and community more frequently.

**Table 1:** Distribution of depression score with CES-DC (19,20) scale by select socio-demographic and risk factors among institutionalized orphaned children in Hyderabad

**Variable**	**Categories**	**Children orphaned by AIDS (COA)**			**Children orphaned by other reasons (COO)**		
		**N = 199**	**Mean**	**Median**	**N = 198**	**Mean**	**Median**
Age*	12 to 14 years	186	34.5	37.0	118	18.4	17.5
	15 to 16 years	13	35.8	41.0	80	23.7	23.0
Sex†	Boy	127	33.2	34.0	80	21.2	22.0
	Girl	72	37.1	43.5	118	20.1	18.5
Type of orphan‡	Maternal orphan	29	36.9	44.0	31	20.9	21.0
	Paternal orphan	114	34.9	39.0	91	19.5	20.0
	Double orphan	56	32.9	34.5	66	21.6	21.0
	Do not know	0	0	0	10	21.8	20.0
Education§	Never been to school/Class 1-5	131	33.7	35.0	44	22.4	22.0
	Class 6-12	68	36.3	40.0	154	20.0	19.0
Religion¶	Hindu	60	32.1	36.5	140	19.7	19.0
	Christian	136	35.5	37.5	43	22.8	24.0
	Others	3	45.0	52.0	12	20.8	20.5
Duration of stay at the orphanage**	<= 2 years	82	33.8	34.0	99	21.6	22.0
	3 - 4 years	81	35.9	40.0	36	18.7	18.5
	> 4 years	36	33.6	35.5	63	20.0	17.0
Ever bullied or ill-treated by friend/relatives††	Yes	100	37.9	40.5	53	25.0	24.0
	No/Cannot say	99	31.2	33.0	145	18.9	18.0
Ever abused at orphanage‡‡	Yes	38	36.3	37.0	46	27.1	27.5
	No	161	34.2	39.0	152	18.6	17.0
Ever witnessed fights between parents§§	Yes	100	36.1	40.0	71	24.9	24.0
	No	70	32.8	34.0	75	17.2	15.0
	Don’t recall/Don’t know	29	33.8	37.0	52	19.4	18.5
Ever experienced discrimination¶¶	Always/many times	25	44.2	46.0	15	26.2	28.0
	Sometimes	50	36.0	40.0	16	27.3	27.0
	Never	124	32.1	34.0	166	19.4	19.0

*p = 0.749 for COA; p = 0.001 for COO.

† p = 0.066 for COA; p = 0.467 for COO.

‡ p = 0.470 for COA; p = 0.636 for COO.

§ p = 0.241 for COA; p = 0.194 for COO.

¶ p = 0.150 for COA; p = 0.227 for COO.

**p = 0.113 for COA; p = 0.325 for COO.

†† p = 0.001 for COA; p < 0.001 for COO.

‡‡ p = 0.419 for COA; p < 0.001 for COO.

§§ p = 0.318 for COA; p < 0.001 for COO.

¶¶ p < 0.001 for COA; p = 0.001 for COO.

ANOVA test for significance is reported.

**Figure 1:**
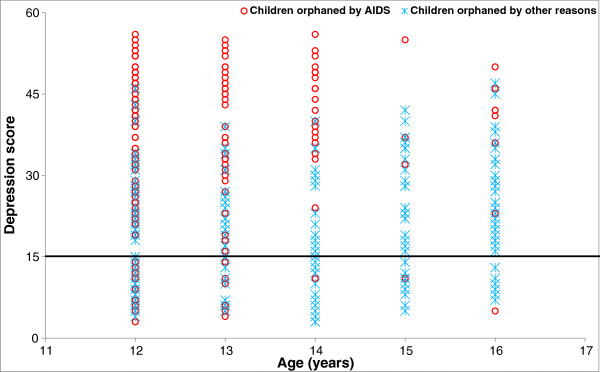
Distribution of depression score with CES-DC (19,20) scale by age for institutionalized orphaned children in Hyderabad.

**Figure 2:**
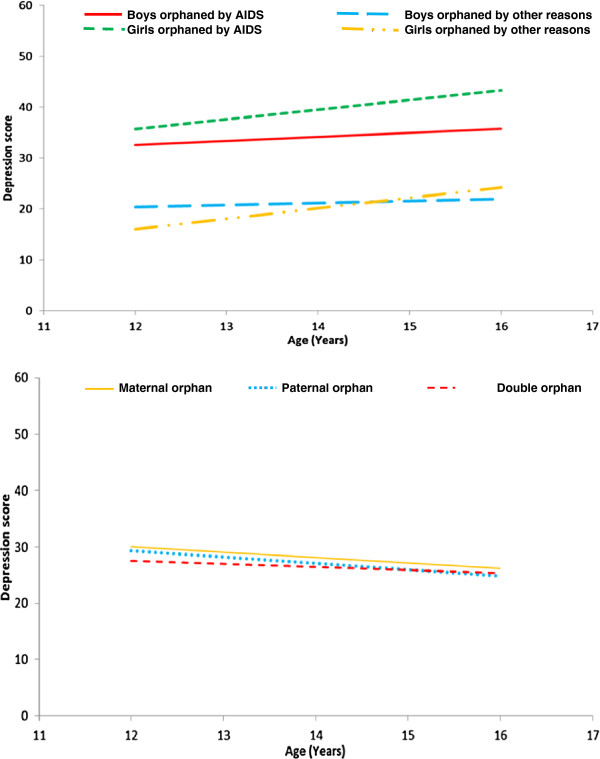
Distribution of depression score with CES-DC (19,20) scale for age by sex and type of orphan among institutionalized orphaned children in Hyderabad.

Among the COA, bulk of the depression score spread was clustered in the younger age groups whereas in the COO it was clustered in the older age groups (p = 0.001) (Figure 1). Increase in depression score was seen with increasing age for girls in both groups but not for boys (Figure 2), however this was not significant. The distribution of depression score declined very gradually with increasing age based on the type of orphans, and these distributions were similar for all the three groups, and the association was not significant.

Table 2 shows distribution of items on the CES-DC scale for the study participants. Overall, the items that were reported the by the majority were: I felt like I was worse than the other kids (a lot, 43.1%), I felt sad (a lot, 40.7%), I felt like crying (a lot, 39.6%), I felt scared (a lot, 33.6%), I felt down and unhappy (a lot, 32%), and I was bothered by things that usually don’t bother me (a lot, 28.5%). COA were more likely to report “feeling sad, feel like crying, feeling scared, bothered by things that usually do not bother them, being let down or unhappy, and feeling worse than the other kids” as compared with the COO. Boys were more likely to report that it was hard to get start doing things (a lot, 27.1%), feel like thing done before did not work out right (a lot, 25.1%), and being too tired to do things (a lot, 22.2%), whereas more girls reported feeling like crying (a lot, 41.3%), feeling let down and unhappy (a lot, 34.7%) and not feel like eating (a lot, 25.5%).

**Table 2:** Contribution of CES- DC (19,20) items towards the burden of depression among the orphaned children in Hyderabad, Andhra Pradesh

**Item description**	**Children orphaned by AIDS (COA) N (% of total, 199)**				**Children orphaned by other reasons (COO) N (% of total, 198)**			
	**Not at all**	**A little**	**Some**	**A lot**	**Not at all**	**A little**	**Some**	**A lot**
I was bothered by things that usually don’t bother me.	30 (15.1%)	17 (8.5%)	57 (28.6%)	95 (47.4%)	91 (46.0%)	26 (13.1%)	63 (31.8%)	18 (9.1%)
I did not feel like eating, I wasn’t very hungry.	55 (27.6%)	21 (10.6%)	54 (27.1%)	69 (34.7%)	98 (49.5%)	34 (17.2%)	50 (25.3%)	16 (8.1%)
I wasn’t able to feel happy, even when my friends tried to help me feel better.	57 (28.6%)	23 (11.6%)	50 (25.1%)	69 (34.7%)	131 (66.2%)	21 (10.6%)	35 (17.7%)	11 (5.6%)
I felt like I was worse than the other kids.	88 (44.2%)	40 (20.1%)	20 (10.1%)	51 (25.6%)	27 (13.6%)	36 (18.2%)	15 (7.6%)	120 (60.6%)
I felt like I couldn’t pay attention to what I was doing.	45 (22.6%)	23 (11.6%)	52 (26.1%)	79 (39.7%)	97 (49.0%)	30 (15.2%)	53 (26.8%)	18 (9.1%)
I felt down and unhappy.	28 (14.1%)	25 (12.6%)	53 (26.6%)	93 (46.7%)	71 (35.9%)	43 (21.7%)	50 (25.3%)	34 (17.2%)
I felt like I was too tired to do things.	36 (18.1%)	37 (18.6%)	62 (31.2%)	64 (32.2%)	120 (60.6%)	26 (13.1%)	40 (20.2%)	12 (6.1%)
I felt like something good was going to happen.	72 (36.2%)	42 (21.1%)	33 (16.6%)	52 (26.1%)	41 (20.7%)	59 (29.8%)	45 (22.7%)	53 (26.8%)
I felt like things I did before didn’t work out right.	41 (20.6%)	28 (14.1%)	63 (31.7%)	67 (33.7%)	108 (54.5%)	20 (10.1%)	50 (25.3%)	20 (10.1%)
I felt scared.*	31 (15.6%)	15 (7.5%)	54 (27.1%)	99 (49.7%)	74 (37.6%)	44 (22.3%)	45 (22.8%)	34 (17.3%)
I didn’t sleep as well as I usually do.*	47 (23.6%)	36 (18.1%)	74 (37.2%)	42 (21.1%)	114 (57.9%)	33 (16.8%)	34 (17.3%)	16 (8.1%)
I was happy.*	53 (26.6%)	35 (17.6%)	51 (25.6%)	60 (30.2%)	69 (35.0%)	51 (25.9%)	54 (27.4%)	23 (11.7%)
I was more quiet than usual.*	38 (19.1%)	42 (21.1%)	62 (31.2%)	57 (28.6%)	102 (51.8%)	40 (20.3%)	36 (18.3%)	19 (9.6%)
I felt lonely, like I didn’t have any friends.*	69 (34.7%)	27 (13.6%)	43 (21.6%)	60 (30.2%)	136 (69.0%)	7 (3.6%)	28 (14.2%)	26 (13.2%)
I felt like kids I know were not friendly or that they didn’t want to be with me.*	66 (33.2%)	28 (14.1%)	41 (20.6%)	64 (32.2%)	140 (71.1%)	17 (8.6%)	25 (12.7%)	15 (7.6%)
I had a good time.*	49 (24.6%)	44 (22.1%)	48 (24.1%)	58 (29.1%)	63 (32.0%)	63 (32.0%)	47 (23.9%)	24 (12.2%)
I felt like crying.*	21 (10.6%)	9 (4.5%)	53 (26.6%)	116 (58.3%)	75 (38.1%)	28 (14.2%)	53 (26.9%)	41 (20.8%)
I felt sad.*	16 (8.0%)	9 (4.5%)	54 (27.1%)	120 (60.3%)	52 (26.4%)	30 (15.2%)	74 (37.6%)	41 (20.8%)
I felt people didn’t like me.*	73 (36.7%)	51 (25.6%)	47 (23.6%)	28 (14.1%)	145 (73.6%)	11 (5.6%)	28 (14.2%)	13 (6.6%)
It was hard to get started doing things.*	40 (20.1%)	28 (14.1%)	60 (30.2%)	71 (35.7%)	121 (61.4%)	28 (14.2%)	37 (18.8%)	11 (5.6%)

*Data not available for one child orphaned due to other reasons other than AIDS.

In MCA (Table 3), the unadjusted means show that the intensity of depression (higher score) was mainly associated with being COA, ever experience of discrimination and being ever bullied by friends/relatives. The adjusted means from MCA analysis showed that being COA (34.4%) had the highest effect on intensity of depression when controlling for all the other variables.

**Table 3:** Multiple classification analysis for effect of socio-demographic characteristics and risk factors for psychological wellbeing on the level of depression in the institutionalized orphaned children in Hyderabad

**Variable**	**Categories**	**N = 386**	**Predicted mean for depression**				
			**Unadjusted mean**		**Adjusted mean**		
			**Mean**	**Eta**	**Mean**	**Beta**	**P value**
Age	12 to 14 years	299	28.3	0.070	27.0	0.101	0.031
	15 to 16 years	87	25.9		30.5		
Sex	Boy	203	28.6	0.059	26.7	0.075	0.087
	Girl	183	26.9		28.9		
Child orphaned by AIDS	Yes	199	34.6	0.484	34.4	0.473	<0.001
	No	187	20.5		20.7		
Type of orphan*	Maternal orphan	60	28.6	0.043	29.2	0.055	0.429
	Paternal orphan	205	28.0		27.9		
	Double orphan	121	26.9		26.8		
Duration of stay at the orphanage	<=2 years	174	27.1	0.161	27.6	0.067	0.283
	3 to 4 years	111	31.2		29.2		
	> 4 years	101	25.1		26.6		
Ever bullied or ill-treated by friend/relatives	Yes	151	33.5	0.315	29.6	0.100	0.055
	No/Cannot say	235	24.1		26.6		
Ever abused at orphanage	Yes	80	31.2	0.121	30.8	0.107	0.016
	No	306	26.9		27.0		
Ever witnessed fights between parents	Yes	167	31.6	0.229	29.6	0.118	0.022
	No	143	24.9		25.7		
	Don’t recall/Don’t know	76	24.8		27.7		
Ever experienced discrimination	Always/many times	40	37.5	0.322	34.2	0.157	0.006
	Sometimes	64	34.0		28.6		
	Never	282	25.0		26.7		
	Full model	386				0.587	<0.001

*11 cases for which data were missing were excluded from the model.

## Discussion

A large proportion of orphaned children reported depression symptoms, and those orphaned due to AIDS experienced more depression than the COO. We examined certain demographic and psycho-social risk factors that may contribute to depression, and being COA had the highest effect on the intensity of depression.

Little is known about the mental health consequences of orphans in India [17,22,26]. The results of this study build upon the prior evidence from other countries of increased vulnerability of orphans and in particular COA [10-13,18,30-35]. Compared to COO, COA were 1.3 times as likely to score ≥15 on the CES-DC scale, a recommended cutoff for clinically significant levels of depressive symptoms [19,20]. Additionally, COA were twice more likely to report experiencing bulling or ill treatment by friends or relatives, a higher proportion had witnessed fights between parents and had experienced discrimination as an orphan. Generally, discrimination has been associated with a number of adverse physical and mental health consequences and therefore, it is important to address stigma and discrimination directed towards children with respect to preventing short term as well as long-term negative health outcomes [36,37]. Factors like discrimination and bullying are addressable at a community level and providing adequate child social support net is considered to be protective during major stressful life events and it has been suggested that it may confer protection with respect to the mental health impact of HIV/AIDS [35,38].

Age and sex of the children were found to be important demographic factors in relation to mental health. A higher mean score for depression was documented in girls orphaned due to HIV/AIDS, younger COA, and in older COO. It is not possible for us to comment on the reasons for such differences as this study was not designed to address these, but knowledge of these differences is important in furthering the understanding of processes to deal with mental health issues in these children. The distribution of items on CES-DC scale was different for boys and girls, and perhaps, can contribute to explanation of the differences in the intensity of depression among boys and girls. Women are known to have higher rates of depression as compared with men [39]. Even though type of orphan hood (maternal or paternal) has been reported to be significantly associated with mental health in children [40], we did not find such an association in our study population.

Some limitations of this study should be taken into consideration. Because of lack of availability of validated mental health scale for children in India, we used CES-DC scale which has been used in various cultures. However, all psychological measures should be interpreted with caution in different cultures. We used continuous scale and not the clinical cut-off score reflecting western norms as it was considered inappropriate for this study population. Another limitation could be misreporting of the parental cause of death, which was documented based on information provided by the child or the NGO staff. It was not possible to confirm the parental cause of death. The cross-sectional nature of these data does not allow temporal or causal explanations as these data do not allow comment on psychological issues in these children prior to them being orphaned. Strength of this study is that this is the largest study to date that has explored psychological issues among COA and COO.

While this study provides evidence of mental health issues in orphaned children, in particular COA, there is lack of specific programme or policy in India to address care of the large number of COA [22,41]. Currently, the only major HIV-related targeted intervention for children in India is HIV prevention from mother-to-child HIV transmission [42]. With India having over 400 million children [43], and an estimated 4.9% with one or both parents dead [44], there is a strong need for interventions targeting the orphaned children. Thought the policy framework in India available for children affected by HIV/AIDS had the mandate to reach at least 80% of the affected children by the year 2010, not much has been achieved [26]. On a positive note, the draft mental health bill in India includes child mental health policy, school mental health policy, and mental health policy for disabled [45]. It is, therefore, important to have an evidence base for understanding the effects of different factors on child mental health for the planned policies and programmes to be effective. As indicated in the integration approach outlined by UNICEF to protect and support children affected by HIV/AIDS [46], an effective national response should provide orphaned children with a package of essential services including education, health care, social welfare and protection; and response at the community level by non-governmental, local and faith-based organizations is suggested. There is also evidence from longitudinal studies in other parts of the world about the continued long term mental health distress among COA as compared with other orphans as they progress through adulthood [47,48]. Therefore, understanding long-term impacts on children of parental death due to a chronic and highly stigmatised disease will assist in formulating effective interventions that reduce such a distress in COA.

## Conclusions

In conclusion, these results suggest that the mental health of COA is significantly worse than that of the COO. It is hoped that these findings can bring more attention from the policy makers and health care providers to the mental health needs of orphaned children, COA in particular.

## Competing interests

The authors declare that they have no competing interest.

## Authors’ contributions

SGPK managed data collection. SGPK and RD wrote the first draft of manuscript. SGPK, GAK and RD performed analysis. All authors contributed to design, interpretation and paper writing, and approved the final version of manuscript.
